# HER2-targeted advanced metastatic gastric/gastroesophageal junction adenocarcinoma: treatment landscape and future perspectives

**DOI:** 10.1186/s40364-022-00416-x

**Published:** 2022-09-30

**Authors:** Weiling Li, Xiaoling Zhang, Yunyi Du, Ying Zhang, Jing Lu, Wenqing Hu, Jun Zhao

**Affiliations:** 1 Department of Oncology, Changzhi People’s Hospital Affiliated to Changzhi Medical College, Changzhi, 046000 Shanxi China; 2 Graduate School, Changzhi Medical College, Changzhi, 046000 Shanxi China; 3 Department of Pathophysiology, School of Basic Medical Sciences, Zhengzhou University, Zhengzhou, Henan 450001 China; 4 Department of Gastrointestinal Surgery, Changzhi People’s Hospital Affiliated to Changzhi Medical College, Changzhi, 046000 Shanxi China

**Keywords:** HER2-targeted therapy, Gastric cancer, Gastric/gastroesophageal junction adenocarcinoma, Monoclonal antibody, Bispecific antibody, Antibody–drug conjugates, Tyrosine kinase inhibitor, Immunotherapy

## Abstract

Recently, the global incidence of gastric/gastroesophageal junction (G/GEJ) cancer has remained high. China is also a large country with a high gastric cancer (GC) incidence rate, where the cases of GC account for 40% of all cases worldwide. More than 90% of GEJ cancers are the adenocarcinoma pathological type. Patients with early-stage G/GEJ adenocarcinoma may have a better prognosis after surgery. In contrast, patients with advanced metastatic G/GEJ adenocarcinoma usually choose comprehensive treatment based on systemic pharmacotherapy, but the subsequent long-term survival is not optimistic. The discovery of various biomarkers, especially microsatellite instability (MSI), programmed cell death-ligand 1 (PD-L1), human epidermal growth factor receptor 2 (HER2), tumor mutational burden (TMB) and Epstein–Barr virus (EBV), has led to the identification of an increasing number of targeted populations and has greatly improved the clinical efficacy of treatments for G/GEJ adenocarcinoma. The ToGA trial added trastuzumab to standard chemotherapy, showed improved survival of patients with HER2-positive advanced G/GEJ adenocarcinoma and brought these patients into a new era of HER2-targeted therapy. Moreover, many HER2-targeted agents have been developed and studied in patients with advanced HER2-positive G/GEJ adenocarcinoma who have demonstrated excellent clinical outcomes. However, many patients experience disease progression with HER2-targeted therapy; hence, new anti-HER2 drugs keep being developed, significantly reducing HER2 resistance. This paper reviews HER2-targeted drugs for advanced metastatic G/GEJ adenocarcinoma, potential resistance mechanisms and future directions.

## Introduction

Gastric cancer (GC) is the fifth most commonly diagnosed cancer worldwide and is the fourth leading cause of cancer-related deaths, with approximately 1 million new cases and approximately 760,000 deaths due to GC worldwide in 2020 [1]. Most patients with nonmetastatic GC select endoscopic or surgical treatment [2], and for patients with advanced metastatic gastric/gastroesophageal junction (G/GEJ) adenocarcinoma, multidisciplinary treatment based on systemic pharmacotherapy is currently recommended. Standard chemotherapy regimens have usually consisted of fluorouracil and platinum with or without paclitaxel, and the resulting long-term survival has not been optimistic [3–5]. Therefore, it is necessary to develop new antitumor drugs and to search for additional therapeutic targets. Human epidermal growth factor receptor 2 (HER2) belongs to the epidermal growth factor receptor (EGFR) family, and its overexpression/amplification has been confirmed in various malignant neoplasms, such as breast cancer (BC), prostate cancer, and lung cancer. The application of HER2-targeted therapy has improved the survival prospects of patients with these malignancies [6–8]. Approximately 7.3–20.2% of patients with advanced G/GEJ adenocarcinoma have HER2 overexpression [9]. The completion of the ToGA trial and the approval of trastuzumab have positively affected the survival of patients with HER2-positive GC, as this drug has become the standard first-line treatment for patients with advanced metastatic HER2-positive GC, which has established the precedent for GC-targeted therapy [10]. The success of the CheckMate-649 trial has opened the door to immunotherapy for advanced GC, which has greatly improved the progression-free survival (PFS) and overall survival (OS) of patients with HER2-negative GC. Nevertheless, in addition to trastuzumab, the following HER2-targeted agents for advanced metastatic G/GEJ adenocarcinoma are still under investigation: monoclonal antibodies (mAbs) (e.g., pertuzumab, margetuximab, hersintuzumab), antibody–drug conjugates (ADCs) (e.g., T-DM1, DS-8201, Disitamab vedotin), bispecific antibodies (BsAbs) (e.g., ZW25, KN026), tyrosine kinase inhibitors (TKIs), and other novel therapeutic approaches (e.g., CAR-T, BVAC-B). In particular, Disitamab vedotin has been approved by the National Medical Products Administration (NMPA) for the second-line and above treatment of patients with HER2-overexpressing advanced metastatic G/GEJ adenocarcinoma. In this review, we summarize the clinical trials and potential resistance mechanisms of HER2-targeted therapy in advanced metastatic G/GEJ adenocarcinoma. We also discuss strategies to overcome resistance to HER2-targeted therapy and the development of new approaches.

## Molecular mechanism of HER2-targeted therapy

### HER2

**Fig. 1:**
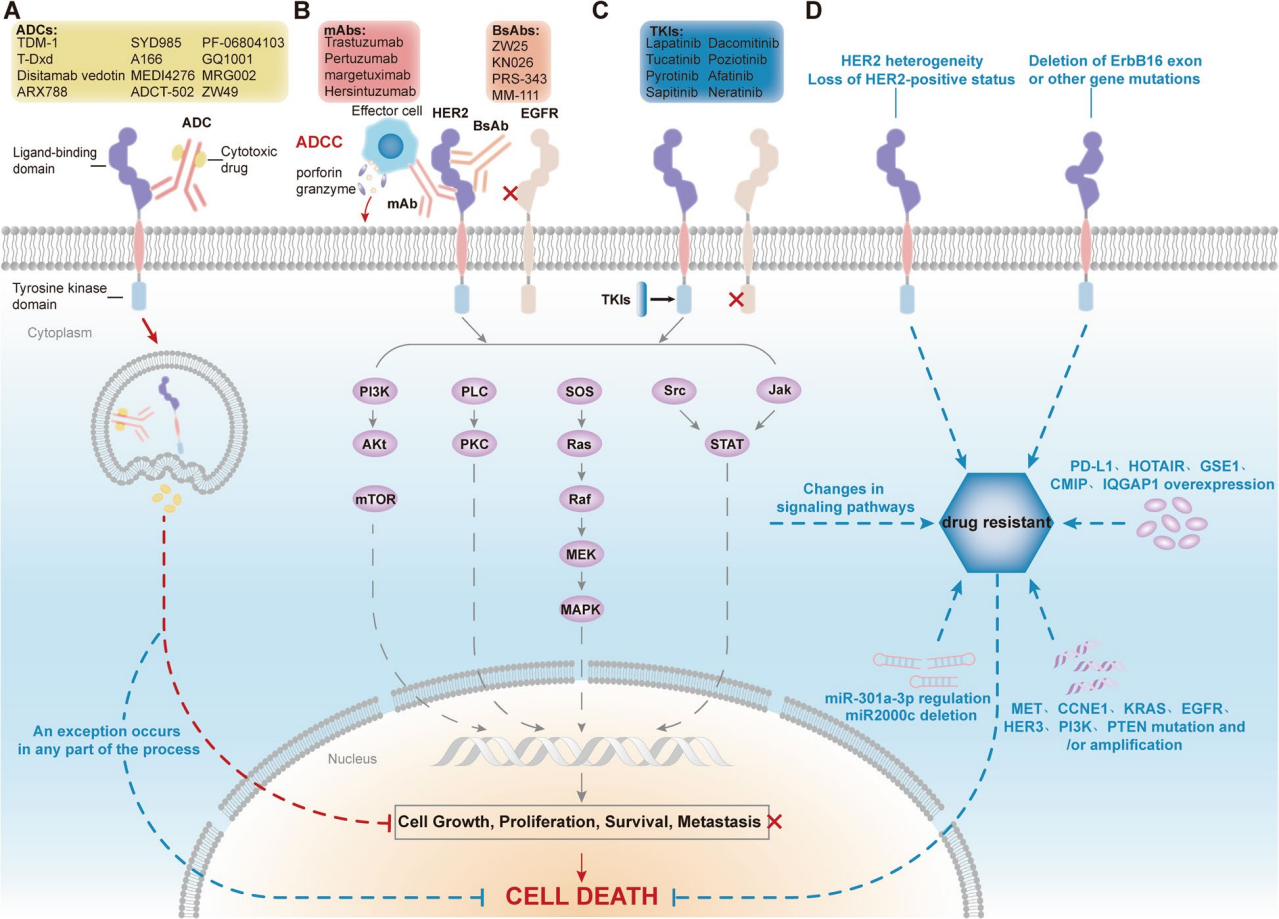
Mechanism of action and potential resistance mechanisms of HER2-targeted therapy: **A** HER2-targeted ADCs enter cells through endocytosis and release toxins that act on microtubules, DNA, or other materials, thereby inhibiting cell growth, proliferation, survival and metastasis. **B** HER2-targeted antibodies inhibit downstream signaling by binding to the extracellular domain of HER2 and preventing the formation of dimers between HER2 and other EGFR family members, in addition to releasing perforins and granzymes to act on target cells through ADCC. **C** TKIs inhibit signal transduction by binding to the intracellular tyrosine kinase domain of HER2. **D** HER2 heterogeneity, loss of HER2-positivity, mutation/amplification, alterations in intracellular signaling, protein overexpression, miRNAs, and abnormalities in either component of the ADC process can all lead to the development of drug resistance, which prevents cell death

HER2, also known as ErbB2/Neu, belongs to the EGFR family and is located on human chromosome 17 (17q21); this gene encodes a 185 kDa transmembrane glycoprotein (p185). EGFR family members include HER1, HER2, HER3, and HER4, which are composed of three parts: an extracellular ligand-binding domain, a transmembrane domain, and an intracellular tyrosine kinase domain. HER2 lacks specific ligands and induces autophosphorylation of intracellular tyrosine residues by forming heterodimers with other members of the family, which activates downstream signaling (e.g., Ras-Raf-Mek-MAPK, PI3K-Akt-mTOR, JAK-STAT); this in turn promotes cell growth and proliferation and inhibits apoptosis [11] (Fig. 1).

### HER2 overexpression/amplification

HER2 overexpression is mainly due to HER2 amplification and leads to constitutive expression of the ERBB signaling network [11]. Overexpression of HER2 results in the formation of homodimers, which in turn results in ligand-independent signaling that leads to uncontrollable cell division, proliferation, differentiation, and apoptosis [11, 12]. HER2 expression is usually detected by immunohistochemistry (IHC) combined with fluorescence in situ hybridization (FISH) according to the following scale: IHC 3+ is HER2-positive and IHC 0/1+ is HER2-negative, whereas IHC 2+ requires FISH and is HER2-positive if FISH amplification is observed and is HER2-negative if FISH amplification is not observed [13]; thus, HER2 overexpression includes IHC 3+ and IHC 2+/FISH+. HER2 overexpression has been observed in BC, bladder cancer, lung cancer and other malignant tumors [6–8]. HER2 overexpression in GC was reported by K. Sakai et al. in 1986 [14]. HER2 positivity was more common in GEJ adenocarcinomas (32.2%) than in GC (21.4%) and was more common in intestinal tumors (31.8%) than in diffuse tumors (6.1%) [15]. In the most recent global report, HER2 overexpression in GC patients accounted for approximately 7.3–20.2% of all cases, and its expression rate varied according to country [9]. A retrospective study of 726 GC cases that underwent surgical resection at 4 clinical centers in China reported a HER2-positive rate of 13% [16], while another global multicenter study evaluated 734 patients with G/GEJ adenocarcinoma at 11 Chinese hospitals and found that 12% were HER2-positive [17].

Furthermore, the relationship between HER2 overexpression and the prognosis of GC patients remains controversial. A prospective study reported a poor prognosis in patients with HER2-overexpressing GC [18], and the results of a multicenter study in Japan that evaluated the relationship between HER2 status and prognosis in 1148 GC patients also showed that HER2 overexpression was associated with prognosis [19]. In contrast Sarah B Fisher et al. analyzed the HER2 status of 111 patients with G/GEJ adenocarcinoma and suggested that HER2 overexpression/amplification was not associated with poor prognosis [20]. Similarly, Shen et al. failed to find an association between HER2 expression levels and prognosis in 1562 GC patients in China [21]. Thus, the prognostic value of HER2 in patients with G/GEJ adenocarcinoma remains unclear and needs to be determined by further studies.

### Low HER2 expression and mutations

Low HER2 expression includes HER2 IHC 1+ and IHC 2+/FISH+ [22]. The CheckMate-649 trial led to the addition of nivolumab to the first-line treatment of patients with HER2-low GC [23], but more response-persistent drugs are still needed. Recent clinical trials have shown that ADCs, such as DS-8201 and Disitamab vedotin, release intracellular toxins that can exert a killing effect on neighboring cells without target expression. This is termed the bystander effect and allows patients with HER2- low GC to benefit from HER2-targeted therapy [24, 25]. Therefore, ADCs may expand the population that can benefit from HER2-targeted therapy and are expected to be a novel option for patients whose tumors have low HER2 expression.

HER2 mutations have been demonstrated in a variety of malignancies, such as lung cancer, colorectal cancer (CRC), BC, and uroepithelial cancer (UC), and are associated with different outcomes in different cancers. Patients with HER2-mutated BC have a worse prognosis than those without mutations; in non-small cell lung cancer (NSCLC), patients with HER2 mutations are more sensitive to some HER2-targeted therapeutic agents. Patients with HER2-mutated CRC also have KRAS mutations, which suggests that concomitant HER2 and KRAS mutations may promote colorectal tumorigenesis, and these patients exhibit little response to EGFR-targeted therapy [26–29]. Several drugs are effective in patients with HER2-mutated NSCLC, such as poziotinib, DS-8201, and T-DM1 [30–32]. Therefore, the detection of HER2 mutations is essential for individualized treatment.

### Molecular mechanism of HER2 blockade

HER2 blockade improves the prognosis of patients with HER2-positive tumors by two primary mechanisms: (1) HER2 blockade prevents the binding of ligands to HER2 receptors and promotes the internalization and degradation of the receptors, which causes inhibition of their downstream signaling pathways, thereby regulating cell survival, proliferation, and invasion [33]; (2) HER2 blockade triggers antibody-dependent cell-mediated cytotoxicity (ADCC) by targeting HER2 receptors, which induces antitumor immunity [34]. Therefore, the development of targeted therapeutics that act on HER2 could be effective in prolonging the survival of patients.

## HER2-targeted therapeutic agents

### HER2-targeted antibodies

#### Monoclonal antibodies (mAbs)

##### Trastuzumab

Trastuzumab is a HER2-targeted mAb that binds to the extracellular domain of HER2 (domain IV) to inhibit downstream signaling and induce ADCC by blocking the activation of the intracellular tyrosine kinase domain [35]. The ToGA trial added trastuzumab to standard chemotherapy, which led to a significant survival benefit for patients with HER2-positive advanced G/GEJ adenocarcinoma (median OS, mOS 13.8 vs. 11.1 months; *P* = 0.046); consequently, trastuzumab in combination with chemotherapy is now the current first-line standard of care for patients with advanced metastatic HER2-positive GC [10]. Subsequent similar trials have yielded positive results and have demonstrated the feasibility of standard-dose trastuzumab in combination with chemotherapy as a first-line treatment regimen in dose-escalation studies [36–41].

Trastuzumab has also shown efficacy as a second-line therapy in untreated HER2-positive GC patients [42]. Moreover, trastuzumab is controversial as a cross-line therapy. A single-center trial in China found that continuation of trastuzumab was effective when progression occurred after first-line standard therapy in patients with advanced GC [43], while the phase II T-ACT trial failed to show improvements in PFS and OS in HER2-positive GC patients who progressed after first-line standard therapy. Notably, this trial did not assess HER2 status prior to continuation of trastuzumab, and in an exploratory analysis, loss of HER2-positive status occurred in 69% (11/16) of evaluable patients after first-line therapy [44].

Trastuzumab has also demonstrated promising utility as a perioperative therapy. Several trials using trastuzumab in combination with chemotherapy or radiotherapy for the perioperative treatment of HER2-positive resectable gastroesophageal adenocarcinoma (GEA) have reported considerable survival benefits [45–47]. The ongoing INNOVATION trial, which is testing trastuzumab or trastuzumab and pertuzumab as a perioperative treatment in patients with HER2-positive resectable G/GEJ adenocarcinoma, indicates that this therapy may be the standard of care in the perioperative period [48]. In contrast, a single-center retrospective study by Qifei He et al. found that trastuzumab added to perioperative therapy in patients with resectable HER2-positive GC resulted in tumor shrinkage and prolonged OS, but no meaningful improvement in OS was observed when trastuzumab was used as a neoadjuvant therapy (*P* = 0.126) [49]. As such, the value of trastuzumab as a neoadjuvant therapy in patients with HER2-positive G/GEJ adenocarcinoma requires further exploration.

##### Pertuzumab

Pertuzumab is a novel humanized anti-HER2 mAb that, unlike trastuzumab, inhibits downstream signaling by binding to the dimer-forming domain of HER2 (domain II) and preventing its dimerization with other EGFR family members [50]. The efficacy of pertuzumab in patients with HER2-positive advanced GC is unclear. In preclinical studies, although pertuzumab plus trastuzumab enhanced antitumor activity [51], the addition of pertuzumab to standard therapy failed to significantly improve the survival of patients with HER2-positive metastatic G/GEJ adenocarcinoma in the first-line placebo-controlled JACOB trial (mOS 17.5 vs. 14.2 months; *P* = 0.056) [52]. The PETRARCA trial evaluated the efficacy of trastuzumab and pertuzumab in combination with chemotherapy for HER2-positive resectable GEA during perioperative care, and although no significant survival benefit was observed, the pathological complete response rate (pCR) was higher in patients who received dual-target therapy [53].

##### Margetuximab

Margetuximab, an Fc-optimized mAb that targets HER2 with the same binding epitope as trastuzumab, affects the cell’s ability to kill tumor cells by increasing the affinity for activated Fc receptor (CD16A) and by decreasing the affinity for inhibitory Fc receptor (CD32B) and ADCC effects [54]. Phase I clinical trials that tested single-agent margetuximab reported favorable tolerability and activity [55]. The CPMGAH22–05 trial tested second-line therapy and beyond in patients with advanced HER2-positive GC, and chemo-free margetuximab plus pembrolizumab led to meaningful outcomes [56]. The ongoing phase II MAHOGANY trial will further explore the efficacy of margetuximab in combination with anti-PD-1 antibody for the first-line treatment of patients with G/GEJ adenocarcinoma [57].

##### Hersintuzumab

Hersintuzumab is a humanized mAb that targets HER2 extracellular domains I-II. In HER2-positive tumor xenograft models, the combination of hersintuzumab and trastuzumab inhibited tumor cell growth and showed the strongest antitumor activity in ovarian cancer and GC xenograft models [58]. This drug is currently being tested in preclinical studies.

#### Bispecific antibodies (BsAbs)

##### Zanidatamab (ZW25)

ZW25 is a novel enzymatic BsAb that targets both HER2 extracellular domains II and IV and activates ADCC. Reliable tolerability and monotherapeutic antitumor activity were demonstrated in a phase I clinical trial [59]. The ongoing phase II trial published data on ZW25 combined with chemotherapy in the first-line treatment of patients with HER2-positive GEA. The study reported a high disease control rate (DCR) of 100% in the ZW25 combined cohort and a reduction in tumor size in all but one patient, but 61% of patients were still receiving treatment at the time of data cutoff (DCR 89%; median PFS, mPFS 12 months) [60]. This finding supports the use of ZW25 in combination with chemotherapy as a potential new first-line standard of care for patients with HER2-positive GEA. Another ongoing trial (NCT04276493) is testing ZW25 in combination with chemotherapy and the anti-PD-1 antibody tislelizumab as a first-line treatment of patients with HER2-positive metastatic G/GEJ adenocarcinoma.

##### KN026

KN026 binds to HER2 domains II and IV to achieve the same effect as the combination of trastuzumab and pertuzumab. In a phase II clinical trial, KN026 showed favorable efficacy in patients with HER2-overexpressing G/GEJ adenocarcinoma with an objective response rate (ORR) of 55.6%; common adverse events included liver dysfunction and rash [61]. The ongoing KN026–001 phase II/III trial will further explore the survival benefit of KN026 in combination with chemotherapy as a second-line and beyond treatment in patients with HER2-positive advanced G/GEJ adenocarcinoma.

##### Other BsAbs

PRS-343 is a BsAb that targets HER2 and the costimulatory immunoreceptor 4-1BB on immune cells. In a phase I clinical trial, PRS-343 demonstrated antitumor activity as a single agent and in combination with the anti-PD-L1 antibody atezolizumab in previously treated patients with advanced HER2-positive solid tumors (including GC) [62]. The phase II trial is currently enrolling (NCT05190445) and will evaluate the efficacy of PRS-343 in combination with ramucirumab and paclitaxel in previously treated patients with treated HER2-overexpressing G/GEJ adenocarcinoma.

MM-111 is a BsAb that specifically binds to HER2 and HER3 and prevents the formation of HER2/HER3 heterodimers. MM-111 was discontinued in a trial of patients with advanced HER2-positive GEA due to worse PFS and OS, and all further clinical trials have now been withdrawn [63]. In addition, several drugs are currently being tested in preclinical studies [64] [65] (Tables 1, 2; Fig. 1B).

**Table 1:** Clinical trials of HER2-targeted antibodies in HER2-positive advanced G/GEJ adenocarcinoma

Drug	Trial/Author Year	Phase Status	Population	Intervention	N	Results	Adverse events
Trastuzumab	ToGA 2010 [10]	III Completed	First-line HER2-positive advanced G/GEJ adenocarcinoma	Trastuzumab + Cisplatin/Fluorouracil Cisplatin/Fluorouracil	298 296	mOS 13.8 m(*P* = 0.0046) 11.1 m mPFS 6.7 m(*P* = 0.0002) 5.5 m	Grade 3/4 68% 68%
	HERBIS-1 2014 [36]	II Completed	First-line HER2-positive advanced GC	Trastuzumab + Cisplatin + S-1	56	mOS 16.0 m mPFS 7.8 m	Grade 3/4 Neutropenia 36% Anorexia 23%
	WJOG7212G 2018 [37]	II Completed	First-line HER2-positive advanced G/GEJ adenocarcinoma	Trastuzumab + Cisplatin + S-1	44	mOS 16.5 m mPFS 5.9 m	Grade 3/4 Neutropenia 30% Anorexia 25%
	CGOG1001 2016 [38]	II Completed	First-line HER2-positive advanced GC	Trastuzumab + Oxaliplatin/Capecitabine	51	mOS 19.5 m mPFS 9.2 m	Grade ≥ 3 Thrombocytopenia 21.6% Anemia 5.9%
	KSCC/HGCSG/CCOG/PerSeUS1501B 2020 [39]	II Completed	First-line HER2-positive advanced/recurrent GC	Trastuzumab + S-1 + Oxaliplatin	42	mOS 27.6 m mPFS 7.0 m	Grade 3/4 Thrombocytopenia 17.9% Anorexia 17.9%
	HERXO 2019 [40]	II Completed	First-line HER2-positive advanced G/GEJ adenocarcinoma	Trastuzumab + Capecitabine + Oxaliplatin	45	mOS 13.8 m mPFS 7.1 m	Grade ≥ 3 Diarrhea 26.6% Nausea 20%
	HELOISE 2017 [41]	IIIb Completed	First-line HER2-positive advanced G/GEJ adenocarcinoma	Standard dose trastuzumab + Capecitabine/Fluorouracil + Cisplatin High-dose Trastuzumab + Capecitabine/Fluorouracil + Cisplatin	33 32	mOS 12.5 m(*P* = 0.2401) 10.6 m mPFS 5.7 m(*P* = 0.8222) 5.6 m	Grade ≥ 3 24.2% 26.8% Grade ≥ 3 59.7% 61.0%
	JFMC45–1102 2016 [42]	II Completed	Second-line HER2-positive advanced GC	Trastuzumab + Paclitaxel	47	mOS 17.1 m mPFS 5.1 m	Grade 3/4 Neutropenia 32.6% Leukopenia 17.4%
	Qian Li 2016 [43]	- Completed	Second-line HER2-positive advanced GC	Trastuzumab + chemotherapy chemotherapy	32 27	mPFS2 3.1 m(*P* = 0.008) 2.0 m mOS2 10.5 m(*P* = 0.172) 6.5 m mOS 22 m(*P* = 0.048) 16 m	Grade ≥ 3 Leukopenia 3 1 Neutropenia 4 3
	WJOG7112G/T-ACT 2020 [44]	II Completed	Second-line HER2-positive advanced G/GEJ adenocarcinoma	Paclitaxel Paclitaxel + Trastuzumab	46 45	mOS 10.0 m(*P* = 0.20) 10.2 m mPFS 3.2 m(*P* = 0.33) 3.7 m	Grade 3 Neutropenia 27% 33% Anemia 24% 31%
	NEOHX 2021 [45]	II Completed	Perioperation HER2-positive resectable G/GEJ adenocarcinoma	Trastuzumab + Capecitabine/Oxaliplatin	36	18 m DFS rate 71% 24 m DFS rate 60%	Grade 3/4 Diarrhea 33% Nausea and emesis 8%
	Ralf-Dieter Hofheinz 2021 [46]	II Completed	Perioperation G/GEJ adenocarcinoma	Trastuzumab + 5-FU + Leucovorin + Docetaxel + Oxaliplatin	56	mDFS 42.5 m 3-year OS rate 82.1%	Grade ≥ 3 Neutropenia 46.6% Infect 21.4%
	NRG Oncology/RTOG 1010 2022 [47]	III Completed	neoadjuvant therapy HER2-positive GEA	Trastuzumab + Paclitaxel + Carboplatin+ Radiotherapy Paclitaxel + Carboplatin + Radiotherapy	102 101	mDFS 19.6 m(*P* = 0.97) 14.2 m	Grade ≥ 3 Hematology system 56% 57% treatment-related death 5% 3%
Pertuzumab	JACOB 2018 [52]	III Completed	First-line HER2-positive metastatic G/GEJ adenocarcinoma	Pertuzumab + Trastuzumab + Cisplatin+ Capecitabine + 5-FU Placebo + Trastuzumab + Cisplatin + Capecitabine + 5-FU	388 392	mOS 17.5 m(*P* = 0.057) 14.2 m mPFS 8.5 m(*P* = 0.0001) 7.0 m	Grade 3–5 Neutropenia 30% 28% Anemia 15% 17%
	PETRARCA 2020 [53]	II terminated	Perioperation HER2-positive resectable GEA	FLOT (paclitaxel + Oxaliplatin + Leucovorin + 5-FU) Pertuzumab/ Trastuzumab + FLOT	41 40	pCR 12%(*P* = 0.02) 35% mDFS 26 m(*P* = 0.14) NA	Grade 3 Diarrhea 5% 41% Leukopenia 13% 23%
ZW25	NCT02892123 2018 [59]	I processing	Second-line and beyond HER2-positive locally advanced/metastatic tumors	ZW25	33(GC 11)	PR 39%(GC 43%) DCR 52%(GC 57%)	Most common Grade 1–2 Diarrhea Transfusion reaction
KN026	NCT03925974 2021 [61]	II Completed	Second-line and beyond HER2-expressing G/GEJ adenocarcinoma	HER2-overexpressing KN026 HER2-expressing KN026	20 11	mOS 11.0 m 9.6 m mPFS 5.6 m 1.4 m	Grade ≥ 3 Transfusion reaction 3.2% High blood pressure 3.2%
PRS-343	NCT03330561 2020 [62]	I Completed	Second-line and beyond HER2-positive GC, other solid tumors	PRS-343	51(GC 19)	DCR 58%	Fatigue 9% Chilliness 6%

*Abbreviations*: *G/GEJ* gastric/gastroesophageal junction, *GC* gastric cancer, *GEA* Gastroesophageal adenocarcinoma, *mOS* median overall survival, *m* months, *mPFS* median progression-free survival, *DFS* disease free survival, *mDFS* median disease-free survival, *pCR* pathological complate response, *PR* partial response, *DCR* disease control rate

**Table 2:** Ongoing and unpublished clinical trials of HER2-targeted therapy

Drug	Trial/Author Year	Phase Status	Population	Intervention	N
Pertuzumab	EORTC-1203-GITCG/INNOVATION 2019 [48]	II processing	perioperation HER2-positive GC	chemotherapy Chemotherapy + Trastuzumab Chemotherapy + Trastuzumab + Pertuzumab	–
ZW25	NCT04276293	Ib/II processing	First-line HER2-positive BC, GC, GEJ adenocarcinoma	ZW25+ Docetaxel ZW25 + Tislelizumab+ Capecitabine + Oxaliplatin	80
KN026	KN026-CSP-001	II/III Recruiting	Second-line and beyond HER2-positive G/GEJ adenocarcinoma	II KN026+ Paclitaxel KN026+ Iirinotecan III KN026+ Paclitaxel + Docetaxel + Irinotecan Placebo + Paclitaxel + Docetaxel + Irinotecan	–
MM-111	Crystal Shereen Denlinger 2016 [63]	II terminated	–	–	–
PRS-343	NCT05190445	II Recruiting	Second-line and beyond HER2-positive GC	PRS-343 + Ramucirumab + Paclitaxel PRS-343 + Tucatinib	80
DS-8201	DESTINY-Gastric03 2020	Ib/II Recruiting	HER2-overexpressing GC	DS-8201 single-agent or combination chemotherapy	255
	DESTINY-Gastric04	III Recruiting	Second-line and beyond HER2-positive metastatic/unresectable G/GEJ adenocarcinoma	DS-8201 Ramucirumab + Paclitaxel	490
	DESTINY-Gastric02	II processing	Second-line and beyond HER2-positive metastatic unresectable G/GEJ adenocarcinoma	DS-8201	79
Disitamab vedotin	NCT04714190	III Recruiting	Second-line HER2-positive locally advanced/metastatic GC	Disitamab vedotin Paclitaxel/Irinotecan/Apatinib	351
ARX788	ACE-Pan Tumor 01	I Recruiting	Second-line and beyond BC, GC, Other solid tumors	ARX788	190
XMT-1522	NCT02952729	Ib completed	Second-line and beyond HER2-positive advanced BC, GC, NSCLC	XMT-1522	120
ZW49	NCT03821233	I Recruiting	Second-line and beyond HER2 positive BC, GEA, other solid tumors	ZW49	174
ADCT-502	NCT03125200	I terminated	Second-line and beyond HER2-positive advanced solid tumors	ADCT-502	21
GQ1001	NCT04450732	I Recruiting	Second-line and beyond HER2 positive BC, GC, other solid tumors	GQ1001	27
MRG002	NCT04492488	I/II Recruiting	Second-line and beyond HER2-positive advanced GC, GEJ adenocarcinoma, other solid tumors	MRG002	129
	CTR20181778 2020	I processing	HER2-positive locally advanced/metastatic solid tumors	MRG002	dose escalation 24 dose expansion 50
Tucatinib	MOUNTAINEER-02/NCT04499924 2021	II/III Recruiting	Second-line and beyond Locally advanced unresectable or metastatic HER2-positive G/GEJ adenocarcinoma	Tucatinib + Trastuzumab + Ramucirumab + Paclitaxel	578
	NCT04430738	Ib/II Recruiting	Second-line and beyond HER2-positive gastrointestinal cancer	Tucatinib + Trastuzumab + FOLFOX	120
	NCT05190445	II Recruiting	Third-line and beyond HER2+ G/GEJ adenocarcinoma	PRS-343 + Ramucirumab + Paclitaxel PRS-343 + Tucatinib	80
	NCT05382364	I Approved	Second-line and beyond HER2+ advanced BC, GC, GEJ cancer, colorectal cancer in China	Tucatinib	25
Pyrotinib	NCT02378389	I completed	Second-line and beyond HER2-positive advanced GC	Pyrotinib/Pyrotinib + Docetaxel	28
	NCT03480256	Id completed	Second-line and beyond HER2-positive advanced GC	SHR6390+ Pyrotinib	40
Afatinib	NCT02501603 2015	II processing	Second-line HER2-positive G/GEJ adenocarcinoma	Afatinib + Paclitaxel	72
	NCT01522768	II processing	Second-line and beyond HER2-positive advanced GEA	Afatinib + Paclitaxel	42
Neratinib	NCT05274048	I Approved	Second-line and beyond HER2-positive GC	Neratinib +DS-8201	18

*Abbreviations*: *GC* gastric cancer, *BC* breast cancer, *GEJ* gastroesophageal junction, *G/GEJ* gastric/gastroesophageal junction, *NSCLC* non-small cell lung cancer, *GEA* Gastroesophageal adenocarcinoma, *FOLFOX* 5-FU + Leucovorin + Oxaliplatin

### Antibody–drug conjugates (ADCs)

#### Trastuzumab emtansine (T-DM1)

T-DM1, an ADC of trastuzumab bound to the antitubulin molecule maytansine, delivers maytansine into tumor cells via receptor-mediated endocytosis, which leads to cell death [66]. T-DM1 inhibits the proliferation of both HER2-positive GC cell lines and trastuzumab-resistant GC cell lines in vivo and in vitro [67]. However, in the phase II/III GATSBY trial, the comparison of T-DM1 and paclitaxel for the second-line treatment of patients with HER2-positive advanced G/GEJ adenocarcinoma failed to show an advantage (mOS 7.9 vs. 8.6 months; *P* = 0.86) possibly because previously archived tumor tissue was selected for this study to assess HER2 status. Those patients had also received HER2-targeted therapy, and thus, HER2 negativity may have led to a lack of response to T-DM1 [68]. The subsequent GATHER3 trial compensated for this disappointing result by including a secondary biopsy in the assessment, and of the 13 HER2-positive GC patients treated with T-DM1, three were negative for HER2 during treatment, and therefore, no disease remission was observed; the ORR of the remaining patients was 44% [69]. This shows the importance of reassessing HER2 status before starting second-line HER2-targeted therapy. Potential drawbacks of T-DM1 have been reported, including a slow internalization rate, insufficient bystander effect, and a lack of intracellular transport and expression of the drug transporter protein MDR1 [70].

#### Trastuzumab deruxtecan (T-Dxd, DS-8201)

DS-8201 is an ADC of trastuzumab coupled to a DNA topoisomerase I inhibitor, which causes cell cycle arrest and apoptosis of tumor cells by binding to topoisomerase I-DNA and inhibiting DNA replication. DS-8201 significantly reduced the risk of death in patients with HER2-positive BC compared with TDM-1 [71], and it was also approved by the U.S. Food and Drug Administration (FDA) in May 2022 for patients with HER2-positive metastatic BC who had previously been treated with anti-HER2 therapy and for those who were treated during or within 6 months of neoadjuvant or adjuvant therapy. In addition, DS-8201 also significantly prolonged PFS and OS in patients with HER2-low BC [22], possibly because the DS-8201 connexon is the enzyme unstable type that facilitates release of the payload after which the nonpolar payload crosses the cell membrane more easily, thus exerting a bystander effect [72]. Moreover, in an animal model study, it was shown that the bystander effect of DS-8201 is dependent on neighboring HER2-positive cells, affects only the tumor microenvironment and does not lead to systemic toxicity [73]. In GC, a phase I trial demonstrated acceptable safety and a favorable response rate [74]. The phase II DESTINY-Gastric01 trial evaluated the efficacy of DS-8201 plus chemotherapy in patients with HER2-positive G/GEJ adenocarcinoma who progressed after first-line treatment. Significant improvements in patient OS (12.5 vs. 8.4 months; *P* = 0.0097) and response rate (51.3% vs. 14.3%) compared with standard therapy were observed, and a survival benefit was also reported in patients with G/GEJ adenocarcinoma with low HER2 expression, with myelosuppression and interstitial lung disease being the major adverse events [24]. Therefore, DS-8201 may provide a new option for patients who progress on prior trastuzumab. In January 2021, the FDA approved DS-8201 for patients with HER2-positive G/GEJ adenocarcinoma who were treated with prior trastuzumab. Other trials are also in progress (NCT04014075, NCT04379596, NCT04704934) [75].

#### Disitamab vedotin

Disitamab vedotin is an ADC consisting of an anti-HER2 mAb and the antitubulin molecule monomethyl auristatin E. This agent has demonstrated safety and potent antitumor activity in a phase I trial in patients with advanced HER2-positive GC [76]. The phase II RC48-C008 trial, conducted in patients with advanced HER2-positive GC for third-line therapy and beyond, also showed a meaningful benefit (ORR 24.8%; mPFS 4.1 months; mOS 7.9 months); the main adverse effects were myelosuppression and malaise [77]. The ongoing phase III RC48-C007 (NCT04714190) trial will further compare the effectiveness of Disitamab vedotin with a standard treatment strategy as a second-line treatment and beyond in patients with advanced HER2-positive GC. In June 2021, Disitamab vedotin was approved in China as a second-line treatment for patients with HER2-overexpressing advanced or metastatic G/GEJ adenocarcinoma. Moreover, a recent preclinical study showed that Disitamab vedotin was more efficacious than trastuzumab in HER2-positive GC patient-derived xenografts (PDX) and demonstrated effectiveness in HER2-negative advanced GC [25].

#### ARX788

ARX788 is a novel ADC formed by the combination of the antitubulin molecule amberstatin 269 and an anti-HER2 mAb; this drug significantly inhibited the growth of tumor cells in a T-DM1-resistant HER2-positive GC model in a preclinical study and was superior to T-DM1 [78, 79]. The phase I ACE-Gastric-01 trial explored the tolerability and antitumor activity of ARX788 in patients with previously treated HER2-positive advanced G/GEJ adenocarcinoma [80]. An ongoing phase I trial will further validate its safety and efficacy (NCT03255070).

#### Other ADCs

With the more prevalent use of ADCs in patients with advanced HER2-positive GC, increasing numbers of drugs have been tested in phase I clinical trials. SYD985 and A166 (NCT03602079) were evaluated in patients with advanced HER2-positive GC and showed preliminary effects [81, 82]. MEDI4276 demonstrated stronger antitumor effects than T-DM1 in vitro, but an objective response was not observed in patients with HER2-positive GC who had previously received standard therapy [83, 84]. The phase 1 trial of ADCT-502 in HER2-positive advanced solid tumors, including GC (NCT03125200), was recently terminated due to safety concerns, and no additional trials are currently underway. PF-06804103 (NCT03284723), XMT-1522 (NCT02952729), GQ1001 (NCT04450732), MRG002 (NCT04492488), and ZW49 (NCT03821233) are also currently being tested in phase I clinical trials [85–88], while several other drugs are being tested in preclinical studies [89]. The development of these new drugs will bring more hope to patients (Tables 2, 3; Fig. 1A).

**Table 3:** Clinical trials of ADCs in HER2-positive advanced G/GEJ adenocarcinoma

Drug	Trial/Author Year	Phase Status	Population	Intervention	N	Results	Adverse events
T-DM1	GATSBY 2017 [68]	II/III terminated	Second-line HER2-positive advanced GC	Docetaxel + Paclitaxel T-DM1 2.4 mg/kg T-DM1 3.6 mg/kg	37 75 70	mOS 8.6 m(*P* = 0.86) 8.6 m 7.9 m	Grade ≥ 3 70% 60%
DS-8201	Kohei Shitara 2019 [74]	I processing	Second-line and beyond HER2-positive advanced G/GEJ adenocarcinoma	DS-8201	44	ORR 43.2%	Grade ≥ 3 Anemia 30% Nneutropenia 20%
	Kohei Shitara 2020 [24]	II Completed	Third-line and beyond HER2-positive advanced G/GEJ adenocarcinoma	DS-8201 Chemotherapy (Irinotecan/Paclitaxel)	125 62(55/7)	ORR 51%(P<0.001) 14% mOS 12.5 m(*P* = 0.01) 8.4 m	Grade ≥ 3 Neutropenia 51% 24% Anemia 38% 23%
Disitamab vedotin	Yingying Xu 2021 [76]	I Completed	HER2-overexpressing locally advanced/metastatic solid tumors	Disitamab vedotin	57	ORR 21.0% DCR 49.1%	Grade ≥ 3 Neutropenia 19.3% Leukopenia 17.5%
	Zhi Peng 2021 [77]	II Completed	Third-line and beyond HER2-overexpressing advanced G/GEJ adenocarcinoma	Disitamab vedotin	125	mOS 7.9 m mPFS 4.1 m	Leukopenia 53.6% Fatigue 53.6%
ARX788	ACE-Gastric-01 2021 [80]	I processing	HER2-positive advanced G/GEJ adenocarcinoma	ARX788	23	ORR 45.5% DCR 50.0%	Grade ≥ 3 26.1%
MEDI4276	Mark D. Pegram 2021 [84]	I Completed	Second-line and beyond HER2-positive BC, GC	MEDI4726	47(32/15)	GC mPFS 1.8 m mOS 6.5 m	Grade 3/4 AST elevation 21.3%
SYD985	NCT02277717 2019 [81]	I Completed	HER2 1+ or higher solid tumors	SYD985	dose escalation 39 dose expansion 146	GC ORR 6%	dose escalation Grade 3/4 keratitis 3 Fatigue 2 dose expansion Fatigue 33% conjunctivitis 31%
A166	NCT03602079 2020 [82]	I-II processing	Second-line and beyond HER2-positive locally advanced/metastatic solid tumors	A166	35	stable disease 33% part response 26% DCR 59%	≥10% Keratitis, decreased appetite, xerophthalmia, blurred vision

*Abbreviations*: *GC* gastric cancer, *G/GEJ* gastric/gastroesophageal junction, *BC* breast cancer, *mOS* median overall survival, *m* months, *ORR* objective response rate, *DCR* disease control rate, *mPFS* median progression-free survival, *AST* aspartate aminotransferase

### Tyrosine kinase inhibitors (TKIs)

#### Lapatinib

Lapatinib, a TKI that targets HER2 and EGFR, blocks autophosphorylation of HER2 intracellular tyrosine kinases, thereby inhibiting downstream signaling. The phase 3 TyTan trial that tested lapatinib as a second-line treatment in Asian patients with advanced GC showed a better survival benefit in the HER2 IHC 3+ subgroup (PFS 5.6 vs. 4.2 months; *P* = 0.0101) but failed to significantly improve OS in the total population (mOS 11.0 vs. 8.9 months; *P* = 0.104), possibly because the overall population of HER2 IHC 0/1+ patients (35%) was higher [90]. Moreover, the TRIO-013/LOGiC trial evaluated the efficacy of lapatinib in combination with standard chemotherapy in patients with HER2-positive advanced GC as a first-line treatment, but lapatinib did not improve patient OS compared with placebo (mOS 12.2 vs. 8.9 months; *P* = 0.349); however, OS was significantly prolonged in Asian and younger patients (< 60 years) in an exploratory analysis [91]. Although lapatinib was beneficial in HER2 IHC 3+ patients and in some Asian and younger patients, these trials did not meet the primary endpoint; thus, lapatinib is not recommended for patients with HER2-positive advanced GC, and its use may require a more rigorous screening strategy. In addition, lapatinib combined with chemotherapy has shown efficacy as a neoadjuvant therapy in patients with resectable HER2-positive GEA; in those patients, diarrhea was a common adverse event but did not interfere with surgical treatment [92].

#### Tucatinib

Tucatinib is a reversible HER2-targeted small-molecule TKI. Tucatinib plus trastuzumab has shown inhibition in HER2-positive GC xenograft models [93], and a phase Ib/II trial of tucatinib combined with trastuzumab and chemotherapy for patients with untreated advanced GC is ongoing (NCT04430738). As a second-line treatment of HER2-positive G/GEJ adenocarcinoma, trials of tucatinib in combination with several agents are currently recruiting patients (NCT04499924, NCT05190445), and the results of a phase I trial of tucatinib alone have just been published (NCT05382364) [94].

#### Pyrotinib

Pyrotinib is a novel oral TKI that targets EGFR, HER2, and HER4. A trial of pyrotinib combined with chemotherapy in patients with advanced HER2-overexpressing solid tumors included 9 patients with GC, whose outcomes were not promising (mPFS: 2.9 months, mOS: 5.9 months) and in whom diarrhea was the most common adverse event [95]. Another phase Ib trial combining pyrotinib with the CDK4/6 inhibitor SHR6390 showed a satisfactory benefit in patients with HER2-positive advanced GC who had received standard therapy, which suggests that this regimen may be a viable treatment strategy for patients with HER2-positive GC, but this requires confirmation in a larger sample [96].

#### Other TKIs

TKIs have been extensively studied in advanced/metastatic HER2-positive G/GEJ adenocarcinoma. The phase II DEBIOC trial of sapitinib in combination with chemotherapy demonstrated antitumor activity in the neoadjuvant treatment of patients with HER2-positive resectable GEA [97]. A phase II trial of dacomitinib monotherapy showed efficacy and safety in patients with HER2-positive GC who had received prior treatment [98]. After poziotinib showed reliable tolerability and toxicity in a phase I trial, a phase I/II trial was conducted in patients with HER2-positive advanced GC, and its combination with paclitaxel and trastuzumab exhibited promising antitumor activity and acceptable toxicity as a second-line therapy [99, 100]. Afatinib showed a favorable benefit in a phase II trial in patients with advanced refractory HER2-positive GEA, and phase II trials of afatinib in combination with paclitaxel as a second-line therapy for advanced/recurrent GEJ adenocarcinoma are ongoing (NCT02501603, NCT01522768) [101]. A phase I clinical trial of neratinib in patients with advanced HER2-positive GC is also ongoing (NCT05274048) (Tables 2, 4; Fig. 1C).

**Table 4:** Clinical trials of TKIs in HER2-positive advanced G/GEJ adenocarcinoma

Drug	Trial/Author Year	Phase Status	Population	Intervention	N	Results	Adverse events
Lapatinib	TyTAN 2014 [90]	III Completed	Second-line HER2-positive GC	Lapatinib + Paclitaxel Paclitaxel	132 129	mOS 11.0 m(*P* = 0.1044) 8.9 m mPFS 5.5 m(*P* = 0.2441) 4.4 m	Grade 3 46% 45% Grade 4 38% 9%
	TRIO-013/LOGiC 2016 [91]	III Completed	First-line HER2-positive advanced GEA	Lapatinib + Capecitabine + Oxaliplatin Placebo + Capecitabine + Oxaliplatin	249 238	mOS 12.2 m(*P* = 0.3492) 10.5 m mPFS 6.0 m(*P* = 0.0381) 5.4 m	Grade 3 Diarrhea 12% 3% Nausea 6% 2%
	Elizabeth C Smyth 2019 [92]	II Completed	Perioperation HER2-positive GEA	Lapatinib + Epirubicin + Cisplatin + Capecitabine Epirubicin + Cisplatin + Capecitabine	22 24	R0 resection 71% 69% Grade 1–2 tumor regression 25% 9%	Neutropenia 53% 54% Grade 3/4 Diarrhea 21% 0
Pyrotinib	Yuzhen Yin 2020 [95]	- Completed	HER2-positive advanced solid tumors (except BC)	Pyrotinib/ + Capecitabine + Paclitaxel + Irinotecan + Pemetrexed + Osimertinib + Bevacizumab + Trastuzumab	25(GC 9)	mOS 9.6 m(GC 5.9个月) mPFS 3.5 m(GC 2.9个月)	Grade 3 Diarrhea 20%
Sapitinib	Anne Thomas 2020 [97]	I Completed	neoadjuvant therapy HER2-positive resectable GEA	Sapitinib + Oxaliplatin + Capecitabine Oxaliplatin + Capecitabine	24	6-m PFS rate 85% 100% 12-m OS rate 80% 100%	Grade 3–4 50% 10%
Dacomitinib	Do-Youn Oh 2016 [98]	II Completed	Second-line and beyond HER2-positive GC	Dacomitinib	27	mOS 7.1 m mPFS 2.1 m	Grade 3/4 Erythra 7% Diarrhea 4%
Poziotinib	Tae Min Kim 2018 [99]	I Completed	Second-line and beyond HER2-positive advanced solid tumors	intermittent Poziotinib continuous Poziotinib	55 20	DCR 63% 53% mPFS 12w 9.0w	Grade ≥ 3 Diarrhea 15% 15% Erythra 2% 0
Afatinib	Francisco Sanchez-Vega 2019 [101]	II Completed	Second-line and beyond HER2-oerexpressing advanced GEA	Afatinib Afatinib + trastuzumab	20 12	mPFS 2 m mOS 7 m	Grade 2 Diarrhea 0 42%

*Abbreviations*: *GC* gastric cancer, *GEA* Gastroesophageal adenocarcinoma, *BC* breast cancer, *mOS* median overall survival, *m* months, *mPFS* median progression-free survival, *DCR* disease control rate

### Combined immunotherapy

In recent years, the emergence of immune checkpoint inhibitors (ICIs) has opened new avenues for GC patients.

Pembrolizumab is a humanized mAb that inhibits PD-1 activity by binding to the PD-1 receptor on T cells. It has been shown that HER2-targeted therapy may increase PD-L1 expression on tumor cells, which would further enhance the potential synergy between these agents [102, 103]. Two clinical trials that tested first-line pembrolizumab in combination with trastuzumab and chemotherapy both showed good survival benefits [102, 104]. The ongoing phase 3 KEYNOTE-811 trial that compared pembrolizumab and placebo to chemotherapy combined with trastuzumab as a first-line treatment for patients with advanced G/GEJ adenocarcinoma has now reached the secondary endpoint with an impressive disease response (ORR 74.4% vs. 51.9%, DCR 96.2% vs. 89.3%) [105]. Based on this trial, the FDA granted accelerated approval of pembrolizumab plus trastuzumab combination chemotherapy in May 2021 for the first-line treatment of patients with locally advanced unresectable or metastatic HER2-positive G/GEJ adenocarcinoma. Moreover, a study of pembrolizumab plus margetuximab for second-line and beyond treatment in patients with advanced HER2-positive G/GEJ adenocarcinoma confirmed the synergistic antitumor effects of HER2-targeted agents and ICIs [56]. Another trial evaluating margetuximab plus the ICIs retifanlimab or tebotelimab with or without chemotherapy for the first-line treatment of patients with HER2-positive G/GEJ adenocarcinoma is underway [57].

Nivolumab is also a humanized mAb that inhibits PD-1 and that has demonstrated efficacy as a third-line therapy and beyond in patients with advanced G/GEJ adenocarcinoma who have been treated with trastuzumab [106]. A phase II trial of nivolumab and trastuzumab in combination with chemotherapy or other ICIs has been completed and is awaiting publication [107]. In addition, the PD-L1/CTLA-4 inhibitor BsAb KN046 in combination with the HER2-targeted BsAb KN026 also showed efficacy in HER-positive solid tumors [108].

Furthermore, the combination of ADCs and ICIs demonstrated antitumor activity in preclinical studies [109]. In the phase Ib DS8201-A-U105 trial, DS-8201 plus nivolumab did not demonstrate a better benefit than DS8201 monotherapy in patients with HER2-positive BC, but the results in the UC cohort were surprising (DCR 76.6%; mPFS 6.9 months; mOS 11.0 months) [110]. The RC48-C014 trial used Disitamab vedotin and toripalimab to treat patients with metastatic UC and showed a survival benefit regardless of whether the patients’ tumors expressed HER2 or PD-L1 [111]. No trials have tested the combination of ADCs and ICIs for advanced GC, and the combination regimen of ADCs and ICIs is inconclusive and requires further confirmation in additional clinical trials (Table 5).

**Table 5:** Clinical trials of combined immunotherapy in HER2-positive advanced G/GEJ adenocarcinoma

Trial/Author Year	Phase Status	Population	Intervention	N	Results	Adverse events
Janjigian YY 2020 [102]	II Completed	First-line HER2-positive G/GEJ adenocarcinoma	Pembrolizumab + Trastuzumab + Chemotherapy	37	mOS 27.3 m mPFS 13.0 m	Grade 3 49% Grade 4 8%
PANTHERA 2020 [104]	Ib-II Completed	First-line HER2-positive advanced GC	Pembrolizumab + Trastuzumab + chemotherapy	43	mOS 19.3 m mPFS 8.6 m	Grade 3 76.7% Grade 4 4.7%
KEYNOTE-811 2021 [105]	III processing	First-line HER2-positive advanced GC	Pembrolizumab + Trastuzumab + chemotherapy Placebo + Trastuzumab + chemotherapy	133 131	RR 74% 52%	–
ATTRACTION-2 2020 [106]	III Completed	Third-line and beyond HER2-positive G/GEJ adenocarcinoma	Nivolumab Placebo	59 22	mOS 8.3 m(*P* = 0.0006) 3.1 m mPFS 1.6 m(*P* = 0.0111) 1.5 m	–
INTEGA/AIO STO 0217 2020 [107]	II processing	First-line HER2-positive advanced/metastatic GEA	Ipilimumab + Nivolumab + Trastuzumab 5-FU/Leucovorin + Oxaliplatin + Nivolumab + Trastuzumab	–	–	–
KN026–203 [108]	II processing	Second-line and beyond HER2-positive G/GEJ adenocarcinoma, BC, other solid tumors	KN046 + KN026	24	ORR 55.0% DCR 85.0% 6-month PFS rate 84.1%	Grade ≥ 3 16.7%

*Abbreviations*: *G/GEJ* gastric/gastroesophageal junction, *GC* gastric cancer, *GEA* Gastroesophageal adenocarcinoma, *BC* breast cancer, *mOS* median overall survival, *m* months, *mPFS* median progression-free survival, *RR* response rate, *ORR* objective response rate, *DCR* disease control rate

### Anti-HER2 chimeric antigen receptor (CAR) cellular therapies and other novel treatments

Anti-HER2 CAR cellular therapies include CAR-T-cell therapy, CAR-natural killer cell (NK) therapy, and CAR-macrophage (CAR-M) therapy. CAR-T-cell immunotherapy integrates the extracellular antigen-binding domain and transmembrane costimulatory domain of tumor-associated antigens (TAAs) with activated T cells, which are expanded in vitro and then infused back into the body to act on tumor cells, causing their death. CAR-T cells can persist in vivo for a long time and can produce durable tumor cell recognition and clearance effects [112]. Yanjing Song et al. found that CAR-T cells produced effective and durable antitumor effects in HER2-positive GC xenograft models [113]. This finding suggests that HER2-targeted CAR-T-cell therapy is a potential therapeutic strategy for patients with HER2-positive advanced GC, but this treatment approach still requires validation in future trials. Several phase I trials evaluating the safety, tolerability and antitumor activity of CAR-T cells in patients with relapsed or refractory HER2-positive solid tumors are currently recruiting patients (NCT04511871, NCT04650451). With the success of CAR-T-cell therapy, Xian Wu et al. introduced HER2-specific CAR (5.137.z) into NK-92 cells, which are termed NK-92/5. 137. z cells; these cells cleared tumor cells in HER2-positive GC xenograft models but did not effectively control larger tumors, which were improved by the addition of apatinib [114]. In addition, CAR-M therapy significantly reduced tumor load and prolonged OS in mouse xenograft models [115]. CT-0508 is a newly developed CAR-M therapy that has shown a survival benefit in a preclinical study of HER2-positive solid tumors and is currently being tested in a phase I clinical trial in patients with HER2-positive solid tumors (including GC) that have progressed after anti-HER2 therapy; the disclosure of the efficacy data is highly anticipated (NCT04660929). A phase I trial of MCY-M11, another CAR containing CAR-Ms, in patients with recurrent/refractory ovarian cancer and peritoneal mesothelioma was terminated due to a shift in focus by the sponsor (NCT03608618). The widespread use of HER2-targeted CAR cell therapy in solid tumors will lead to new drug candidates for patients with HER2-positive solid tumors.

Other novel therapeutic approaches include B-cell and monocyte-based immunotherapeutic vaccines (BVAC-B), ultrasound-mediated sonodynamic therapy, BAY2701439 and CAM-H2 targeted HER2 radiotherapy (NCT04147819, NCT04467515) [116, 117].

## Potential resistance mechanisms of HER2-targeted therapy

The ToGA trial has brought GC into the era of HER2-targeted therapy, and increasing numbers of HER2-targeted therapeutics have been widely studied in GC, yet most patients with advanced GC still inevitably experience disease progression or death due to resistance. Primary or acquired resistance is a major challenge for GC patients, and multiple potential resistance mechanisms have been explored (Fig. 1D).

### HER2 heterogeneity

HER2 heterogeneity includes changes in HER2 expression status and HER2 copy number. During HER2-targeted therapy, tumor cells with HER2 overexpression or HER2 amplification die, while residual drug-resistant cells proliferate and gradually dominate, which eventually leads to tumor recurrence. The heterogeneity of HER2 expression has been reported to be high (45–79%) in HER2-positive GC and is significantly correlated with patient survival; thus, the heterogeneity of HER2 expression was considered a factor in resistance to HER2-targeted therapy [118–121]. In addition, heterogeneity in HER2 expression is observed between primary and metastatic sites, which leads to an increased risk of false-positive HER2 failure of HER2-targeted therapy [122, 123].

### Loss of HER2-positive status

In some clinical trials, 29–69% of GC patients may experience loss of HER2-positive status after trastuzumab progression, which is an important cause of resistance [44, 69, 118]. Therefore, the HER2 status of patients should be re-evaluated upon progression after HER2-targeted therapy to select the optimal treatment.

### Mutation/amplification

Mutations are also a potential cause of resistance to HER2-targeted therapy. De novo mutations in HER2, which are located in the part of the protein involved in the regulation of kinase activity, have been identified in trastuzumab-treated HER2-overexpressing GC cell lines and lead to the maintenance of the active structure of HER2 by affecting its conformation [124]. Several studies have also reported that deletion of ErbB16 exons and comutation and/or amplification of MET, CCNE1, KRAS, EGFR, HER3, PI3K or PTEN can also lead to the development of resistance [101, 118, 125].

### Alterations in intracellular signaling

HER2-targeted therapy inhibits the transduction of downstream signaling by blocking the binding of HER2 receptors and ligands, which inhibits the migration and proliferation of tumor cells and leads to apoptosis. Alterations in receptor tyrosine kinase-RAS-PI3K signaling have been reported to be associated with acquired resistance to trastuzumab [118]. Additionally, activation of the bypass pathway can lead to resistance. In their study, Aïda Sampera et al. found that the development of drug resistance in HER2-positive GC cell lines was associated with sustained activation of the MAPK-ERK and PI3K-mTOR pathways mediated by SRC [126]. In addition, NRF2 leads to the development of drug resistance by activating PI3K-mTOR [127].

### Protein overexpression

Overexpression of some proteins leads to the development of resistance. High expression of PD-L1 was found in trastuzumab-resistant HER2-positive GC cells, and blockade of PD-L1 reversed trastuzumab resistance in trastuzumab-resistant cells. Preliminary results from the KEYNOTE-811 trial also confirmed the synergistic effect of trastuzumab combined with the anti-PD-L1 antibody pembrolizumab [105, 128]. Overexpression of HOTAIR, GSE1, CMIP, and IQGAP1 in GC cells also enhanced resistance to trastuzumab [124, 129–131].

### MicroRNAs (miRNAs)

miRNAs are usually exosome-rich and can spread between cells and exert regulatory effects on drug resistance in various cancers. miR-301a-3p is induced by endoplasmic reticulum stress and mediates trastuzumab resistance by regulating the expression of multiple proteins in HER2-positive GC cells [132]. miR200c deletion induces trastuzumab resistance through TGF-β [133].

### ADC-specific resistance mechanisms

After ADC enters the body and binds to the target antigen on the surface of tumor cells and is endocytosed by tumor cells, the ADC binds to the Fc receptor in the endosome and is then transported to the cell surface for release into the extracellular space. However, other ADC-antigen complexes enter the lysosome, where enzymes or the acidic environment can degrade the ADC, thus releasing cytotoxic chemicals that play a role in tumor cell death. Abnormalities in either component of this process can lead to the development of drug resistance. Preclinical studies have shown that altered internalization and transport pathways and abnormal metabolism are associated with T-DM1 resistance in HER2-positive GC cell lines, but these findings have not been confirmed in a clinical setting [134, 135].

## Future prospects

The advent of HER2-targeted agents offers new hope for patients with advanced HER2-positive GC. The ToGA trial enabled the addition of trastuzumab to the first-line standard of care for patients with advanced HER2-positive GC and ushered in a new era of HER2-targeted therapy, while the benefits of trastuzumab as a neoadjuvant and adjuvant therapy have also been demonstrated. However, HER2-targeted agents other than trastuzumab, such as pertuzumab, T-DM1, and lapatinib, have failed to demonstrate better efficacy as a first-line therapy. Dual HER2-targeted therapy also did not significantly improve the prognosis of patients with advanced GC. Therefore, HER2-targeted therapy still needs to be improved. Several trials have revealed synergistic effects of HER2-targeted therapy combined with anti-PD-L1 antibody in HER2-positive advanced GC. With the approval of combination chemotherapy regimens consisting of pembrolizumab and trastuzumab, many researchers have also begun to experiment with other combinations of HER2-targeted agents and ICIs for advanced HER2-positive GC. This shows that the first-line treatment of advanced/metastatic G/GEJ adenocarcinoma has entered a new era of immunotherapy in combination with targeted therapy and chemotherapy. In addition, in G/GEJ adenocarcinoma with low HER2 expression (HER2 IHC 1+ and IHC 2+/FISH-), ADCs (e.g., Disitamab vedotin, DS-8201) use cleavable connexons that can release payloads before internalization, some of which are hydrophobic or nonpolar payloads that can easily cross cell membranes. They also demonstrate excellent ability through bystander effects but may also cause toxicity in nontumor tissues, and thus, this therapeutic approach is still not sufficiently mature; consequently, more targeted drugs with good safety and durable response profiles are urgently needed (Table 6).

**Table 6:** Current approved drugs and NCCN 2022 recommended drugs for patients with advanced/metastatic GC

Drug	Population	Treatment Setting	Approved/Time	NCCN2022 Recommendation
Trastuzumab	HER2-positive metastatic GC	First-line	FDA/2010.10 NMPA/2012.08	First-line therapy for patients with HER2-positive advanced/metastatic GC
Disitamab vedotin	HER2-overexpressing (IHC 2+/3+) advanced/metastatic G/GEJ adenocarcinoma	Second-line and beyond	NMPA/2021.06	/
Pembrolizumab	HER2-positive locally advanced unresectable/metastatic G/GEJ adenocarcinoma (PD-L1 CPS ≥ 1)	First-line	FDA/2021.5	First-line therapy for patients with HER2-positive locally advanced/metastatic G/GEJ adenocarcinoma Second-line and beyond for dMMR, MSI-H or TMB-H tumors
DS8201	HER2-positive locally advanced/metastatic G/GEJ adenocarcinoma	Second-line and beyond	FDA/2021.01	Second-line and beyond for patients with HER2-positive locally advanced/metastatic G/GEJ adenocarcinoma
Nivolumab	Advanced/metastatic G/GEJ adenocarcinoma	First-line	FDA/2021.04 NMPA/2021.08	HER2-first-line therapy for patients with advanced/metastatic GC
Apatinib	HER2-positive Advanced G/GEJ adenocarcinoma	Third-line	NMPA/2014.12	/

*Abbreviations*: *GC* gastric cancer, *G/GEJ* gastric/gastroesophageal junction, *PD-L1* programmed cell death-ligand 1, *CPS* combined positive score, *FDA* the U.S. Food and Drug Administration, *NMPA* national medical products administration, *dMMR* mismatch repair-deficient, *MSI-H* high microsatellite instability, *TMB-H* high tumor mutational burden

Trastuzumab resistance is also a major obstacle in the treatment of GC patients. Loss of HER2-positive status after disease progression with HER2-targeted therapy has been observed in several studies. Therefore, it is necessary to reassess HER2 status in patients who require later-line therapy.

In conclusion, HER2 is a promising therapeutic target, and although many HER2-targeted drugs are available, the treatment efficiency in patients with advanced GC is low, and the survival benefit is not satisfactory due to the high heterogeneity of GC and the development of resistance. Therefore, new HER2-targeted drugs should continue to be developed to improve the survival of GC patients.

## Data Availability

The material supporting the conclusion of this review has been included within the article.
